# Steam Reforming of Model Bio-Oil Aqueous Fraction Using Ni-(Cu, Co, Cr)/SBA-15 Catalysts

**DOI:** 10.3390/ijms20030512

**Published:** 2019-01-25

**Authors:** José A. Calles, Alicia Carrero, Arturo J. Vizcaíno, Lourdes García-Moreno, Pedro J. Megía

**Affiliations:** Chemical and environmental group, Rey Juan Carlos University, c/Tulipán, s/n, 28933 Mostoles, Spain; alicia.carrero@urjc.es (A.C.); lourdes.garcia@urjc.es (L.G.-M.); pedro.megia@urjc.es (P.J.M.)

**Keywords:** hydrogen production, hydroxyacetone, acetic acid, phenol, furfural, nickel, coke, thermodynamic equilibrium

## Abstract

Hydrogen obtained from biomass derivatives is considered a promising alternative to fossil fuels. The aim of this work is to test the viability of Ni-M/SBA-15 (M: Co, Cu, Cr) catalysts for the hydrogen production from bio-oil aqueous fraction reforming. Tests were performed in a fixed-bed reactor at 600 °C and atmospheric pressure. Firstly, the steam reforming (SR) of acetic acid, hydroxyacetone, furfural and phenol, as representative constituents of the bio-oil aqueous fraction, was carried out. Lower reactivity with increasing carbon number and decreasing steam-to-carbon ratio was observed. Coking rate during SR is a consequence of carbon number and aromaticity of the reactant, as well as the steam-to-carbon ratio. However, deactivation also depends on the graphitization degree of carbon filaments, higher in the case of coke formed from phenol. Then, the performance of the Ni-M/SBA-15 catalysts was studied in the reforming of a bio-oil aqueous fraction surrogate containing the four model compounds. Ni-Co/SBA-15 and Ni-Cr/SBA-15 samples were the most active because Co also catalyze the steam reforming reactions and Cr promotes the formation of very small Ni crystallites accounting for high conversion and the low coke deposition (~8 times lower than Ni/SBA-15) in the form of poorly condensed carbon filaments.

## 1. Introduction

The continuous increase of energy demand based on fossil fuels has caused what is known as an energy problem, mainly characterized by [1,2,3]: (i) Limited energy resources: Society may face the premature depletion of fossil fuels. (ii) Economic factors: The disproportionate increase experienced in its extraction and use involves a continuous and irreversible trend in the growth of the costs of fossil fuels. (iii) Environmental impact: Negative environmental effects of global scope are linked to processing and consumption of energy from fossil fuels, which can be summarized in acid rain, climate change and destruction of the ozone layer, as well as acidification of soil and waters.

The international scientific community agrees that it is necessary to seek greater energy efficiency and energy models not based on fossil fuels. Therefore, increased diversification of sources, renewable energy and improving energy efficiency are major concerns [4,5,6].

Currently, research is guided to seek alternative energy resources that can replace existing sources completely or in part, provided they are clean, renewable and profitable [2,7]. Among these, biofuels and hydrogen have been highlighted to replace fossil fuels [3,8,9]. At the present time, hydrogen is considered an alternative fuel and its use is becoming more important. Hydrogen production has current interest in fuel cell applications, automobile applications and the production of electricity. Hydrogen is also used as a raw material in chemical synthesis and refining for the production of clean fuels. Currently, 97% of hydrogen production is achieved by steam reforming of natural gas and other fossil fuels [9]. However, different products derived from biomass, such as ethanol, glycerol or bio-oils, are alternatives for the production of hydrogen by steam reforming of oxygenated compounds [10,11,12,13,14]. The diversity of sources to obtain hydrogen makes it a promising energy vector and allows its production almost anywhere in the world.

In this context, bio-oils are a dark brown organic liquid from the pyrolysis of biomass, which contain numerous and complex oxygenate organic compounds, such as acids, alcohols, aldehydes, ketones, phenols and other oxygenates derived from biomass carbohydrates and lignin [15,16]. Bio-oils can be separated into two fractions: a water-insoluble phase and an aqueous phase. While the insoluble fraction can be used as a fuel and/or for the production of chemicals, the aqueous phase does not have many applications and it is discarded in most cases. This aqueous fraction contains water soluble oxygenates in total content between 15 and 60 wt %, depending on the feedstock, operating conditions and the catalyst of the pyrolysis process. These factors also determine the necessity of water addition for phase separation [16,17]. Therefore, the catalytic steam reforming of this aqueous fraction of bio-oils could be an interesting way to valorize it and benefit the economics of bio-oil production [18].

Stoichiometrically, the steam reforming of oxygenates in the bio-oil aqueous fraction can be represented as follows:

CH_x_O_y_ + (2−*y*) H_2_O → CO_2_ + (*x*/2+2−*y*) H_2_ (1)

The process is characterized by additional difficulties derived from the extremely heterogeneous composition of the bio-oil aqueous phase. This is the reason why researchers tend to use model compounds to study this process [19,20,21,22,23,24]. Among these, hydroxyacetone, acetic acid, phenol and furfural are good candidates, since acids are 19–25 wt %, ketones are 12–20 wt %, phenols are 1–5 wt % and furans are around 1 wt % of bio-oil [25], and thus they are generally major compounds in the aqueous fraction [26].

Steam reforming catalysts are usually based on supported nickel, but the main drawback of these materials is catalyst deactivation by carbon deposition. In order to reduce it, addition of promoters has been previously described in the literature. One strategy is the increase of the support basicity by adding alkaline (Li, K, Na) or alkaline-earth (Mg, Ca) elements, in order to prevent carbon formation favored on acidic sites [17,27,28,29,30,31,32]. Lanthanides (La, Ce) have also been reported to inhibit the carbon deposition on reforming catalysts’ surface [17,32,33,34,35]. Another approach to improve steam reforming catalysts is the incorporation of a second transition metal. In this sense, Ni–Cu bimetallic catalysts have been extensively studied in ethanol steam reforming, showing that CO production and coke deposition are decreased with the addition of certain loadings of Cu [36,37,38]. Several studies [39,40,41,42] have shown that Ni–Co bimetallic catalysts are able to significantly decrease carbon deposition during the steam reforming reaction, and they have also increased activity and selectivity to H_2_ in steam reforming of oxygenates compounds. On the other hand, Cr addition has been claimed to be beneficial to reduce coke formation reactions [43,44] and inhibit the encapsulation of the nickel by carbon filaments [45], as well as suppress sintering of the active sites by dilution of the ensembles of Ni atoms [46,47], which in turn increases the stability of nickel catalysts.

Since the metal distribution over the support is important to obtain good conversion and selectivity to hydrogen, the choice of supports with highly developed surfaces to promote dispersion of the active metals is considered to be promising. Wang et al. [21] showed how the use of Al_2_O_3_ nano-rods as the support of Ni catalysts instead of commercial alumina led to superior performance in terms of activity and stability during steam reforming of bio-oil model compounds, attributed to higher metal dispersion. Yang et al. [48] demonstrated higher catalytic activity and stability of Ni/meso-MgO catalyst compared to conventional Ni/MgO. This is due to smaller and uniform Ni nanoparticles thanks to the high surface area and the confinement effect of the mesoporous structure of meso-MgO, which could effectively limit the growth of the active metal and stabilize Ni particles during the procedure of NiO reduction. SBA-15 is a mesostructured silica material with high specific surface area, narrow pore size distribution, and high thermal and hydrothermal stability, which has also exhibited enhanced reforming activity by improving Ni dispersion [36,37].

We recently demonstrated the benefits of adding a second metal to Ni/SBA-15-based catalysts on glycerol steam reforming [49]. Particularly, the novel Ni-Cr/SBA-15 catalyst exhibited considerably higher glycerol conversion and hydrogen production than the Ni/SBA-15 material with a drastic reduction in coke formation. However, to date, the reforming of the bio-oil aqueous fraction has not been studied on this kind of material.

Thus, the aim of this work is of this work is to test the viability of Ni-M/SBA-15 (M: Co, Cu, Cr) catalysts for the hydrogen production from bio-oil aqueous fraction reforming. Firstly, these catalysts have been tested in the steam reforming of single model compounds representative of the constituents of the bio-oil aqueous fraction— acetic acid, hydroxyacetone, furfural and phenol—with the aim of comparing how the reforming of each of these compounds proceeds, in terms of the products distribution (including coke formation) and conversion. Then, the prepared catalysts have been tested in the reforming of a bio-oil aqueous fraction surrogate containing the four model compounds, and the effect of the promoters’ incorporation on the catalytic activity has been studied.

## 2. Results and Discussion

### 2.1. Characterization of Fresh Catalysts

The actual Ni loading and textural properties, mean Ni particle size, and reducibility properties of the fresh catalysts are summarized in Table 1. Detailed characterization of these materials has been published elsewhere [49]. Briefly, N_2_-adsorption analyses revealed that all materials exhibit type IV isotherms, typical of the pore mesostructure of the SBA-15 material used as the support. Incorporation of the second metal (Cu, Co or Cr) to Ni/SBA-15 just slightly decreased surface area (calculated according to Brunauer–Emmett–Teller—BET), pore volume and pore diameter. Only peaks corresponding to metallic Ni could be observed in the X-ray diffraction (XRD) patterns of all catalysts after reduction and the mean crystallites diameter (D_Ni_) calculated by applying the Scherrer equation is summarized in Table 1. It must be taken into account that, when applying the Scherrer formula to the calculation of the average crystallite size, from the full width at half maximum intensity (FWHM), smaller crystallites, which contribute mainly to the wings of the line profile, are scarcely taken into account and D_Ni_ could be overestimated. The extreme limit of size that can be measured by XRD is 2 nm [50]. In any case, the addition of Cu, Co and Cr to Ni/SBA-15 helps to form smaller Ni crystallites; the smallest are found in the Ni-Cr/SBA-15 sample.

**Figure 1:**
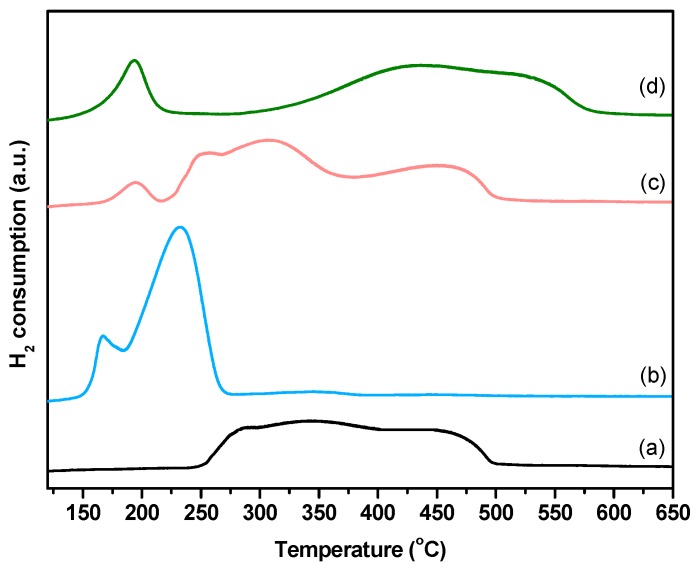
H_2_-TPR profiles of calcined catalysts: (**a**) Ni/SBA-15; (**b**) Ni-Cu/SBA-15; (**c**) Ni-Co/SBA-15; (**d**) Ni-Cr/SBA-15.

From hydrogen temperature-programmed reduction H_2_-TPR analysis of the calcined samples (Figure 1), several reduction features are distinguised, which could be divided into two main regions: a low-temperature zone (below 400 °C) that can be assigned to the reduction of metal oxide particles weakly interacting with the SBA-15; and a high-temperature zone (above 400 °C), which includes the reduction of metal oxide species with strong interaction with the support. The contribution of each region to the reduction profile, obtained after deconvolution, is collected in Table 1. While Ni-Co/SBA-15 showed a similar reduction profile to Ni/SBA-15, with around 30% of the reduction taking place above 400 °C, the Ni-Cu/SBA-15 sample could be almost completely reduced below 400 °C. On the contrary, the main reduction of the Ni-Cr/SBA-15 catalyst (83.5%) takes place above 400 °C and a higher temperature is needed to completely reduce it in comparison to the rest of samples. According to H_2_-TPR results, particles of Ni species strongly interacting with the support predominate in Ni-Cr/SBA-15 catalyst.

### 2.2. Steam Reforming of Bio-Oil Aqueous Fraction Model Compounds

#### 2.2.1. Thermodynamic Analysis of Steam Reforming of Model Compounds 

**Figure 2:**
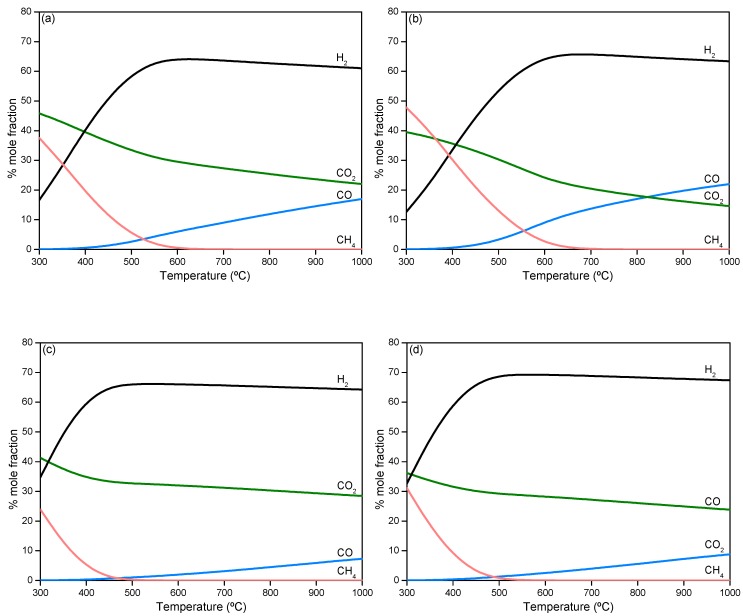
Thermodynamic prediction of equilibrium products’ distribution with temperature in the steam reforming of acetic acid (**a**), hydroxyacetone (**b**), furfural (**c**) and phenol (**d**).

According to thermodynamic predictions, the complete conversion of each of the model compounds through steam reforming is reached at equilibrium in the whole temperatures range from 300 to 1000 °C. Figure 2 shows the equilibrium composition of the outlet stream (dry basis) when reforming is carried out with a water/model compound mixture similar to that experimentally used. Only H_2_, CO_2_, CO and CH_4_ are present in the gas phase. No formation of molecules with two or more carbons (C_2+_) was predicted although side reactions may occur, since the energy of those molecules is generally higher than the energy of the final state, which is highly unstable under steam reforming conditions. Therefore, C_2+_ hydrocarbons are intermediate products in the reaction scheme which are absent in the reaction product stream at equilibrium conditions. This coincides with previous studies [51,52,53].

According to this, from the thermodynamics point of view, the steam reforming of the bio-oil aqueous fraction compounds can be represented by a combination of the following equations:

Steam reforming: CH_x_O_y_ + (1−*y*) H_2_O → CO + (*x*/2+1−*y*) H_2_ (2)

Water-gas shift: CO + H_2_O → CO_2_ + H_2_ (3)

Methanation: CO + 3H_2_ → CH_4_ + H_2_O (4)

CO_2_ + 4H_2_ → CH_4_ + 2H_2_O (5)

Regarding the evolution of products with temperature, in all cases, the hydrogen content in the outlet stream increases with temperature, as the methane content decreases, to reach a maximum. This is caused by steam reforming (Equation (2)) being favored as temperature increases, because this is an endothermal reaction while methanation reactions (Equations (4) and (5)) are unfavored, as they are highly exothermal. Since water-gas shift (Equation (3)) is an exothermal reaction, the CO mol % increases at the expense of CO_2_ as the temperature increases, consuming also H_2_ (reverse reaction). This leads to the decrease of the H_2_ content above a temperature at which methanation is almost completely unflavoured (CH_4_ content below 0.2 mol %).

The maximum H_2_ production at equilibrium, as well as the temperature at which it is reached, depends on the compound fed to reforming and the steam/carbon ratio (S/C). The highest H_2_ content in the product stream (69.2 mol %) is reached by phenol steam reforming at 565 °C. According to the stoichiometry of the global reforming reaction of each of the compounds (Equations (6)–(9)), 70 mol % H_2_ could be reached by phenol and hydroxyacetone SR, while 66.7 mol % H_2_ would be expected for acetic acid and furfural SR.

Acetic acid SR: C_2_H_4_O_2_ + 2H_2_O → 2CO_2_ + 4H_2_ (6)

Hydroxyacetone SR: C_3_H_6_O_2_ + 4H_2_O → 3CO_2_ + 7H_2_ (7)

Furfural SR: C_5_H_4_O_2_ + 8H_2_O → 5CO_2_ + 10H_2_ (8)

Phenol SR: C_6_H_6_O + 11H_2_O → 6CO_2_ + 14H_2_ (9)

The fact that lower H_2_ content is achieved with hydroxyacetone than phenol or even furfural is due to the lower S/C fed to the reactor, which limits steam reforming and water–gas shift reactions and favors methanation. In addition, temperature at which maximum H_2_ production takes place is in the range 550–700 °C, the lowest temperature corresponding to phenol and furfural SR due to the high S/C. On the basis of these results, a temperature of 600 °C was selected to perform the catalytic tests using the Ni-M/SBA-15 catalysts. The same temperature was used for both the steam reforming of model compounds and the simulated bio-oil aqueous phase in order to extract valuable conclusions regarding the interaction between the different reactants in the mixture.

#### 2.2.2. Steam Reforming of Model Compounds on Ni-M/SBA-15 Catalysts

**Figure 3:**
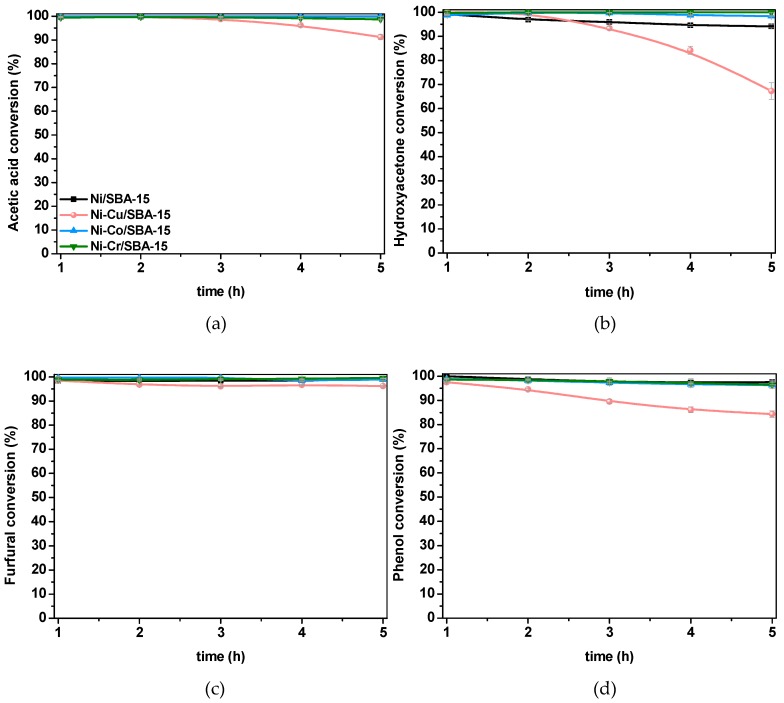
Evolution of conversion with time during the steam reforming of acetic acid (**a**), hydroxyacetone (**b**), furfural (**c**) and phenol (**d**) over Ni-M/SBA-15 catalysts.

**Figure 4:**
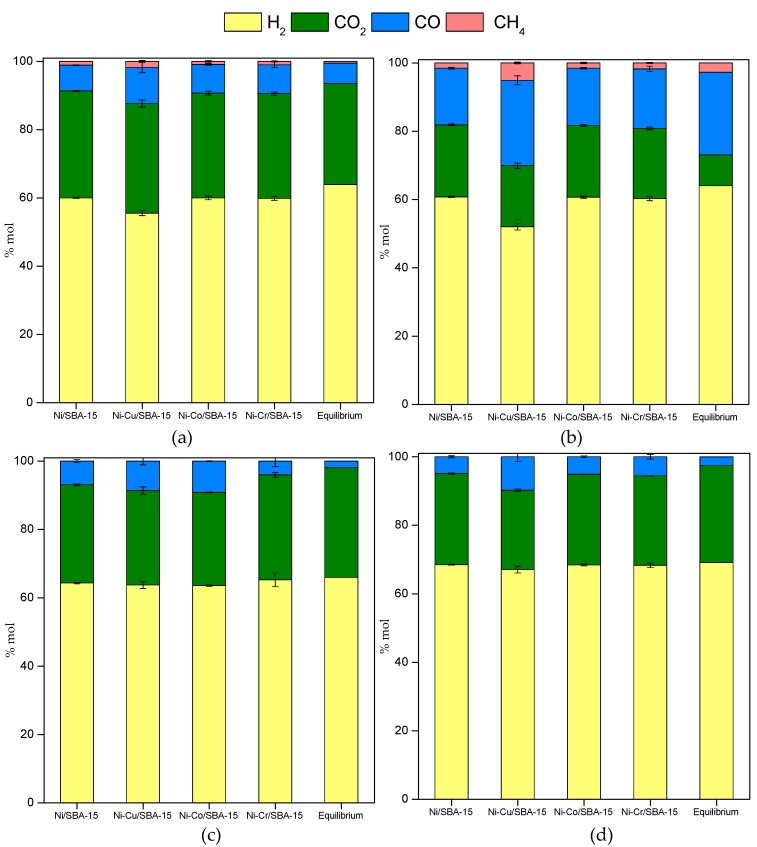
Gaseous products distribution (dry basis) from steam reforming of (**a**) acetic acid, (**b**) hydroxyacetone, (**c**) furfural and (**d**) phenol over Ni-M/SBA-15 catalysts.

The results obtained in the steam reforming of the different model compounds using the prepared catalysts are shown in Figure 3 and Figure 4, in terms of conversion and product distribution in the gas stream, respectively. In all cases, the tested catalysts kept conversions above 95 % under the present operation conditions, except for the Ni-Cu/SBA-15 sample, which clearly suffers from deactivation. In general, conversions were higher in the steam reforming of acetic acid (AA) and furfural (Fur), while the steam reforming of hydroxyacetone (HA) led to lower conversion, coinciding with the highest loss of activity of the Ni-Cu/SBA-15 sample. Ni-Co/SBA-15 and Ni-Cr/SBA-15 were the most active samples. This may be ascribed to the smaller Ni crystallites’ size, especially in the Ni-Cr/SBA-15 sample (see Table 1), and the presence of Co, which is known for its reforming activity [11], in the Ni-Co/SBA-15 catalyst. Comparing feedstocks with similar S/C ratio, in order to exclude the positive effect of water excess, AA (S/C = 4) is more easily reformed than HA (S/C = 2.67) while furfural (S/C = 13.2) is easier to convert than phenol (S/C = 11). This indicates a decrease in reactivity with increasing carbon number, in agreement with Trane-Restrup et al. [54]. Although the C–C bonds in aromatic compounds (Fur and Ph) are more stable than in AA or HA, which may result in lower reactivity [21], the higher S/C ratio used with those promoted higher conversion.

The main products obtained in the gas stream were H_2_, CO_2_, CO and CH_4_ (Figure 4). Only acetone could be detected as an intermediate in the condensate stream of AA and HA steam reforming but with concentrations lower than 0.1 wt % and 0.7 wt %, respectively. This intermediate could be formed by ketonization of acetic acid or hydrodeoxygenation of hydroxyacetone, but its low concentration indicates that it was almost fully converted to hydrogen and carbon oxides, because as predicted by thermodynamics (Section 2.2.1), it should be highly unstable under our reaction conditions.

In the case of AA steam reforming, the hydrogen content in the gas stream ranges between 55 (Ni-Cu/SBA-15) and 60 mol % (Ni/SBA-15, Ni-Co/SBA-15 and Ni-Cr/SBA-15), relatively close to the equilibrium value, 63.9 mol %. CH_4_ formation is low (<1.8 mol %), but slightly higher than the equilibrium value, while CO and CO_2_ are the main C_1_ products with CO_2_/CO ratios below equilibrium. This may indicate that both CH_4_ and CO are intermediates in the AA steam reforming pathway, while CO_2_ should be a final product. This can be explained by decomposition (Equation (10)) or decarboxylation (Equation (11)) of AA, followed by methane steam reforming (Equation (12)) and water–gas shift (Equation (5)), taking place in a more complex pathway than the general one proposed from the thermodynamic analysis (Equations (2)–(5)), where CH_4_ was formed from the products of steam reforming through methanation reaction unfavored at high temperatures.

Decomposition of AA: CH_3_COOH → 2CO + 2H_2_ (10)

Decarboxilation of AA: CH_3_COOH → CH_4_ + CO_2_ (11)

Methane SR: CH_4_ + H_2_O → 3H_2_ + CO (12)

Decarboxylation and decomposition are side reactions based on a mechanism of dissociative adsorption of AA on the catalyst to form acetate (CH_3_COO*) and/or acyl (CH_3_CO*) species, which decompose to lead to the formation of methyl species (CH_3_*; *x* ≤ 3) with release of CO_2_ and CO, respectively. Depending on the reaction conditions (temperature, S/C ratio, H_2_ concentration, etc.) and the catalyst surface, the methyl species can either be hydrogenated to CH_4_ or cleave off further H atoms (CH_3_* → CH_x_*; *x* < 3) [55,56]. The remaining carbon would react with hydroxyl groups from the dissociative adsorption H_2_O on the catalyst, forming H_2_ and CO; otherwise, carbon deposition would occur. According to our results, only a small fraction of methyl species is hydrogenated to CH_4_ under the present conditions. Instead, steam reforming reactions are favored on the Ni/SBA-15-based catalysts at 600 °C and S/C ratio = 4.

The CO_2_/CO ratio lower than equilibrium would confirm that CO is the primary product of steam reforming reactions, further being converted into CO_2_ by water–gas shift. While the rest of samples achieved CO_2_/CO ratios higher than 4.0, the less active Ni-Cu/SBA-15 sample led to a CO_2_/CO ratio lower than 3.4. In addition, for this catalyst, a trace amount of acetone (less than 0.1 wt %) was detected in the liquid outlet stream, indicating that ketonization of acetic acid (Equation (13)) also occurred during the steam reforming process. Acetone can further undergo steam reforming (Equation (14)) or, on the contrary, it can polymerize by aldol condensation to form coke deposits (Equation (15)). Although dehydration to ketene (Equation (16)) has also been described to occur during AA steam reforming, no evidence of such reaction was observed in this work, which may be ascribed to the low acidity of these catalysts, since dehydration is usually favored on acidic materials [57]. The absence of ketene in the gas phase products implies that ketene is a surface intermediate readily reacting further [55], probably through steam reforming (Equation (17)).

Ketonization of AA: 2CH_3_COOH → CH_3_COCH_3_ + CO_2_ + H_2_O (13)

Acetone steam reforing: CH_3_COCH_3_ + 2H_2_O → 3CO + 5H_2_ (14)

Acetone polymerization: CH_3_COCH_3_ → oligomerization → coke (15)

Dehydration of AA: CH_3_COOH → CH_2_CO + H_2_O (16)

Ketene steam reforming: CH_2_CO + H_2_O → 2CO + 2H_2_ (17)

Since ketonization and decomposition reactions occur in parallel with the steam reforming, the hydrogen production is lower for the Ni-Cu/SBA-15 catalyst, which shows poorer activity for AA steam reforming. The rest of samples reached similar hydrogen production.

Similar results were obtained in the HA steam reforming, with hydrogen contents in the gas stream between 52 (Ni-Cu/SBA-15) and 61 mol % (Ni/SBA-15, Ni-Co/SBA-15 and Ni-Cr/SBA-15), the equilibrium value being 64.0 mol %. CO and CO_2_ are the main C_1_ products with CO_2_/CO ratios below half the equilibrium value. On the other hand, contrary to the case of AA, the CH_4_ content in the gas phase is slightly lower than equilibrium prediction, except for the Ni-Cu/SBA-15 sample. Again, it can be explained by a reaction network more complex than that extracted from the thermodynamic analysis (Equations (2)–(5)). Methane can be formed by HA decomposition (Equation (18)), maybe occurring in several steps implying some intermediates. Since acetone was detected in the condensate phase, the hydrodeoxygenation of HA (Equation (19)) should be taking place in parallel to steam reforming, and acetone decomposition (Equation (20)) would lead to CH_4_ formation. Although it is accompanied by ketene formation, the fact that ketene was not detected among the products may be explained by its high reactivity, probably through steam reforming according to (Equation (17)).

Ketene steam reforming: CH_2_CO + H_2_O → 2CO + 2H_2_ (17)

Decomposition of HA: CH_3_COCH_2_OH → CH_4_ + 2CO + H_2_ (18)

Hydrodeoxygenation of HA: CH_3_COCH_2_OH + H_2_ → CH_3_COCH_3_ + H_2_O (19)

Acetone decomposition: CH_3_COCH_3_ → CH_2_CO + CH_4_ (20)

According to Wang et al. [56], the decomposition mechanism of HA starts with the formation of CH_3_COCH_2_O* or CH_3_COCH_2_* species. The latter could form acetone by hydrogenation [58]. However, the most likely reaction pathway involves the CH_3_COCH_2_O* species, further decomposing to methyl species (CH_3_*) through several steps implying acyl (CH_3_CO*) species, similarly to AA decomposition but with no expected CO_2_ release. Catalyst and operating conditions would determine CH_4_ formation by CH_3_* hydrogenation or H_2_ and CO generation by cleavage of C–H bonds of CH_3_* and interaction of the remaining carbon precursor with OH* or O* intermediates formed from H_2_O decomposition. In this case, the low CH_4_ content among the gas products and the high carbon oxides formation indicate that these Ni/SBA-15-based catalysts favored steam reforming reactions over decomposition of HA under the present operating conditions. However, poorer activity of Ni-Cu/SBA-15 sample, as shown in Figure 3b, led to higher CH_4_ formation, lower CO_2_/CO ratio and, consequently, lower H_2_ production than the rest of samples.

Finally, in the steam reforming of Fur and Ph, only H_2_, CO_2_ and CO were detected as reaction products. The absence of CH_4_ in the products stream is ascribed to the high S/C ratio and the fact that there is no CH_3_ group in the structure of Fur and Ph [59]. This implies that CO_x_ formation by steam reforming of CH* or C* species formed from the decomposition of the Fur or Ph ring [60] is more favored than the hydrogenation of those intermediate species to CH_4_ in these reaction conditions. In addition, although methanation could take place according to the reactions scheme suggested for the equilibrium calculations (Equations (4) and (5)), this reaction is disfavored at 600 °C due to its exothermicity. Regarding hydrogen production, the equilibrium concentration is nearly reached in both Fur and Ph steam reforming. The slight differences in hydrogen formation are in line with the CO_2_/CO ratio, so that the higher the ratio the higher the hydrogen content among the products. Contrary to the previous feedstocks, despite the lower conversion of the Ni-Cu/SBA-15 catalysts in the Fur and Ph steam reforming (Figure 3), the hydrogen content is also near the equilibrium prediction. This is probably a consequence of the favorable reaction conditions derived from the high S/C ratio used with these reactants as a consequence of their low solubility.

#### 2.2.3. Coke Formation during the Steam Reforming of Model Compounds on Ni-M/SBA-15 Catalysts

All the used catalysts were analyzed by thermogravimetric analyses (TGA) in order to determine coke deposition and coking rates summarized in Table 2. Independently of the model compound fed in the steam reforming test, the coking rate varies in the order: Ni-Cu/SBA-15 > Ni/SBA-15 > Ni-Co/SBA-15 > Ni-Cr/SBA-15.

**Figure 5:**
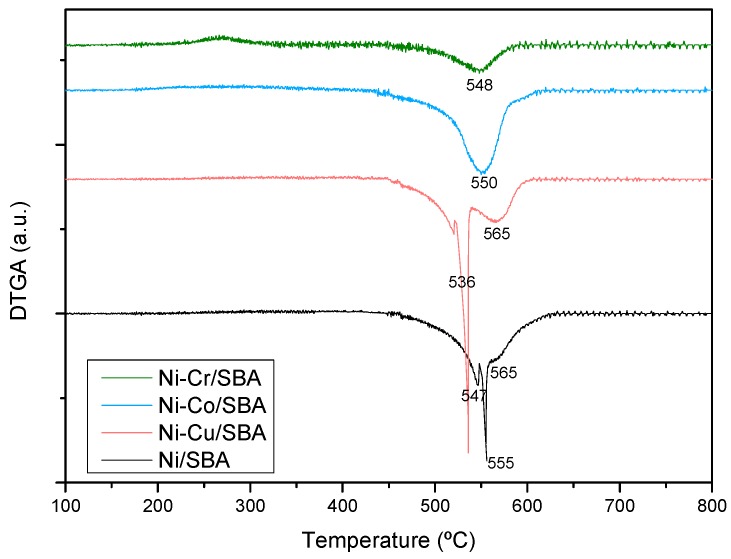
Derivative thermogravimetric analyses (airflow) of the Ni-M/SBA-15 catalysts used in steam reforming of hydroxyacetone.

It is noticeable that the coking resistance of Ni-Cr/SBA-15 decreases by 2 (Ph) to 13 (AA) times the amount of coke formed over our reference material Ni/SBA-15. This is mainly attributed to the small Ni crystallites in this sample which avoid the formation of the intermediary surface species of coke deposition [30]. The presence of Co, which is less prone to coke accumulation than Ni [11], would explain the also high coking resistance of the Ni-Co/SBA-15 sample. On the contrary, enhancement of the carbon formation mechanism has been reported at certain Cu loadings in Cu-Ni catalysts [61], which accounts for the Ni-Cu/SBA-15 sample leading to the highest coke deposition. This sample also showed a decrease of conversion relative to time-on-stream (see Figure 3), which would indicate a relationship between the amount of coke formed and deactivation. Since this effect was more noticeable in the HA steam reforming, the corresponding derivative thermograms are shown in Figure 5. All the samples show a wide Derivative Thermogravimetry (DTG) profile between 450 and 625 °C with maximum carbonaceous matter combustion rate taking place between 536 and 565 °C (T_max_). These temperatures are in accordance with the combustion of filamentous coke typically formed over Ni catalysts during steam reforming [29,30] and the wide profile indicates the presence of carbonaceous species with different ordering degree. Concretely, in the case of Ni/SBA-15 and Ni-Cu/SBA-15 samples, two regions can be distinguished. Peaks at lower temperature correspond to less ordered carbon while those at 565 °C indicate higher graphitization degree of the carbon species. The characteristics of the coke deposited in steam reforming is a result of a balance between its formation and its elimination by gasification, which depends on the operating conditions, time-on-stream and the catalyst employed [62]. As coke is formed, it evolves from amorphous towards filamentous coke, which progressively undergoes condensation, increasing its ordering degree and finally leading to graphitic coke [63], which has been described as the cause of deactivation by blocking the Ni particles. Thus, the peak at lower temperature in the DTG profile of Ni-Cu/SBA-15 may be ascribed to incipient structures of filamentous coke, still present as a consequence of the high carbon formation rate of this catalyst, while the peak at 565 °C may be ascribed to aged filaments in the form of a more graphitic coke, which burns at higher temperature. The presence of this aged coke is the cause for the loss of conversion with time of Ni-Cu/SBA-15 sample. Similarly, the peak of this more graphitic coke can be observed in the profile of Ni/SBA-15 sample, but in lower proportion, which would account for the slight deactivation observed in this catalyst.

**Figure 6:**
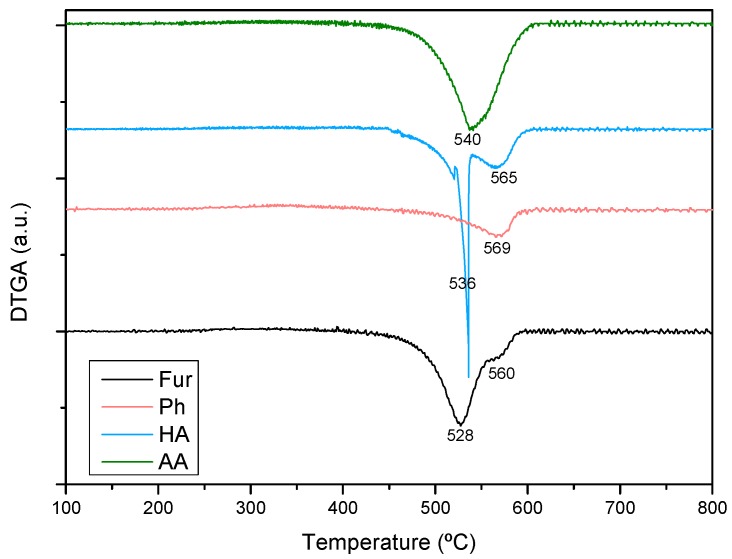
Derivative thermogravimetric analyses (airflow) of the Ni-Cu/SBA-15 catalyst used in steam reforming of different feedstocks.

Regarding carbon deposition from different feedstocks, Table 2 shows that the coking rate varies in the order: HA > AA > Fur > Ph. It has been stated that carbon formation in steam reforming is largest for olefins and aromatics and also larger for large molecules compared with smaller ones [13]. However, coke deposition depends on the S/C ratio since higher steam content in the feed should favour gasification of carbonaceous material. Given the significantly higher S/C ratio used with in Ph and Fur, the higher coking rate in HA and AA steam reforming can be explained. It is interesting to note that, although the lowest coke amount was formed with Ph, deactivation was more severe than that of steam reforming of Fur or even AA. Thus, the nature of coke should depend on the feedstock. Figure 6 shows the derivative thermograms of the used Ni-Cu/SBA-15 sample. The maximum carbon oxidation temperatures (T_max_) correspond to filamentous coke in all cases, but different peaks corresponding to various ordering degrees can be observed depending on the steam reforming feedstock. The peak assigned to a more graphitic coke is the only one that can be observed (T_max_ = 569 °C) in the Ph steam reforming, while the peak assigned to a poorly evolved coke (T_max_ = 528 °C) is predominant in the Fur steam reforming. This could be related with the loss of activity observed with Ph as opposed to Fur steam reforming.

### 2.3. Steam Reforming of Model Bio-Oil Aqueous Fraction

The Ni-M/SBA-15 catalysts were finally tested in the steam reforming of a bio-oil aqueous fraction surrogate at 600 °C. The feed for these tests consisted of an aqueous mixture of the model compounds studied in Section 2.2 simulating the aqueous fraction obtained in the catalytic fast-pyrolysis of de-ashed wheat straw as reported in reference H-ZSM-5 [16]. Typically, bio-oil is recovered as a complex mixture needing water addition for phase segregation and so leading to water contents around 80 wt % in the aqueous fraction [17,64]. In our case, the water content is relatively low (41 wt %) since two phases were directly obtained in the pyrolysis process and could be separated without extra water addition. The advantage of this low water content in the bio-oil aqueous fraction is that the S/C ratio can be adjusted by a simple water addition step. Consequently, the composition of the aqueous fraction used in this work was 31 wt % acetic acid, 18 wt % hidroxyacetone, 6 wt % furfural and 4 wt % phenol in water. The S/C molar ratio corresponding to this feed is 0.95, while the stoichiometric S/C ratio would be 1.28, according to Equation (1). No S/C ratio adjustment was carried out in order to work under harsh operation conditions during the steam reforming, allowing the observation of clear differences in the performance of each catalyst (conversion, product distribution and coke formation) at 5 h time-on-stream (TOS).

**Figure 7:**
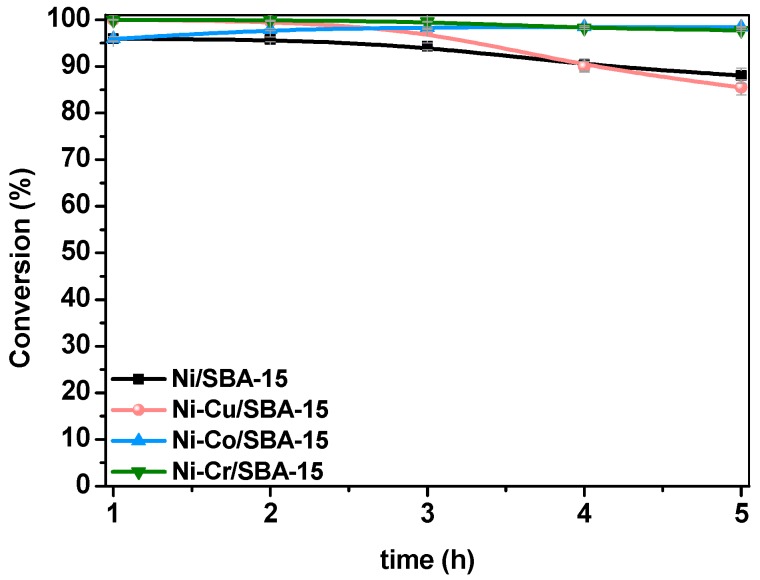
Carbon conversion in the reforming of model bio-oil aqueous fraction over Ni-M/SBA-15 catalysts.

Regarding conversion, Figure 7 displays the evolution with time-on-stream of the carbon conversion (X_C_), calculated from the flowrates of carbon fed (F_C,feed_) and carbon in the condensate product stream (F_C,condensate_), as follows:

(21) $$\mathrm{Xc}\left(\%\right)=\frac{F_{C,feed}-F_{C,\;condensate}}{F_{C,feed}}\cdot 100$$

As expected, differences among the various catalysts are highlighted when using an adverse S/C ratio. While Ni-Co/SBA-15 and Ni-Cr/SBA-15 maintained carbon conversion above 97%, deactivation of Ni/SBA-15 and Ni-Cu/SBA-15 could be observed within 5 h TOS. This result is in line with those obtained in the steam reforming of the single model compounds, mainly in the HA reforming where deactivation could be noticed due to high coke deposition. The reason for the higher activity, as mentioned before, is the presence of Co as an additional active metal in Ni-Co/SBA-15 catalyst and the small Ni crystallites in the Ni-Cr/SBA-15 sample. The improvement of the Ni dispersion by Cr addition has been described to be caused by inhibition of the enlargement of Ni particles during thermal processes (calcination, reduction or even reaction), since chromium oxide can act as a structural promoter [44,46]. Some studies suggest that the Cr species distribute on or surrounding the Ni surface, and thus the Ni particles should be much more difficult to move or sinter under thermal conditions [47,65]. Decrease in size of the Ni crystallites due to the structural promoting effect of chromia induces an increase of the number of such active centers, which in turns increases the reforming activity. In order to analyse the conversion of the model compounds fed toghether in the mixture, the corresponding values are collected in Table 3. Appart from the unconverted fed compounds, acetone was detected in the condensate stream in concentrations between 1.0 and 4.0 wt %, as a consequence of ketonization of acetic acid or hydrodeoxygenation of hydroxyacetone. In general, conversion varies in the order: HA > AA > Fur > Ph. Contrary to the results obtained in Section 2.2, despite its lower carbon number, AA conversion is lower than HA. However, it must be taken into account that acetic acid can be formed as an intermediate during Fur steam reforming [66], which would increase its content in the condensate stream, leading to lower conversion values. With this exception, reforming of the mixture confirms the lower reactivity of molecules with increasing carbon number and aromaticity. 

**Figure 8:**
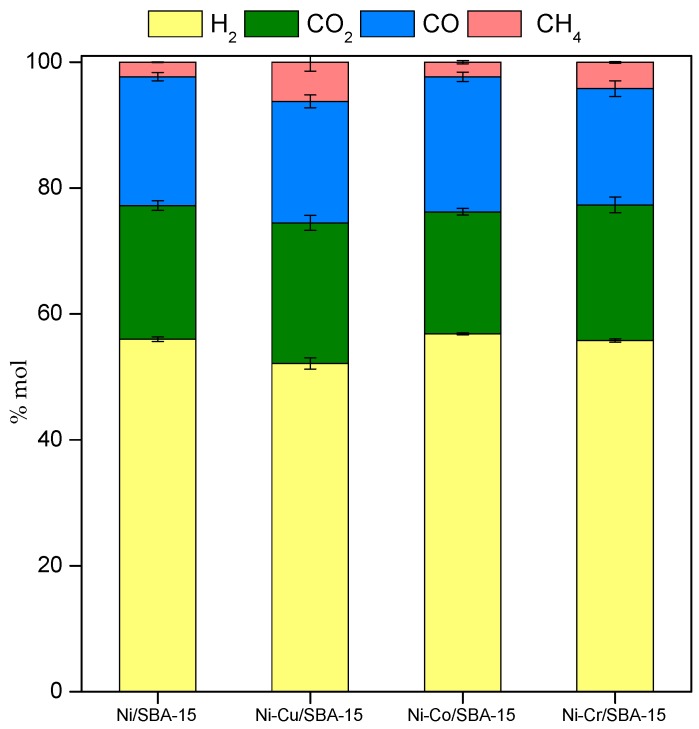
Gaseous products distribution (dry basis) obtained from reforming of model bio-oil aqueous fraction over Ni-M/SBA-15 catalysts.

Product distribution in the gas outlet stream is shown in Figure 8, as mol % in dry basis. Hydrogen content was similar, above 56 mol %, for all catalysts, except in the case of Ni-Cu/SBA-15 sample, which only reached 52 mol %. Compared to the steam reforming of single model compounds, hydrogen production is lower in the reforming of the model bio-oil aqueous fraction since the S/C ratio is lower, which implies less OH* or O* intermediates (formed from water under reaction conditions) available to react with the CH_x_* or C* species. This is supported by the fact that higher CH_4_ amounts are found among the gaseous products in comparison to the steam reforming of the single model compunds. Since hydrogenation of CH_x_* and C* to CH_4_ compete with the reaction of these intermediates with OH* or O*, lower S/C ratio would favour hydrogenation. As a consequence, lower CO_2_/CO ratios are also found in the reactor outlet stream, with values around 1. Comparing catalysts, the lower hydrogen content in the products stream when using Ni-Cu/SBA-15 is accompanied by higher CH_4_ amount, which indicates lower capacity of this sample to activate the water molecules.

**Figure 9:**
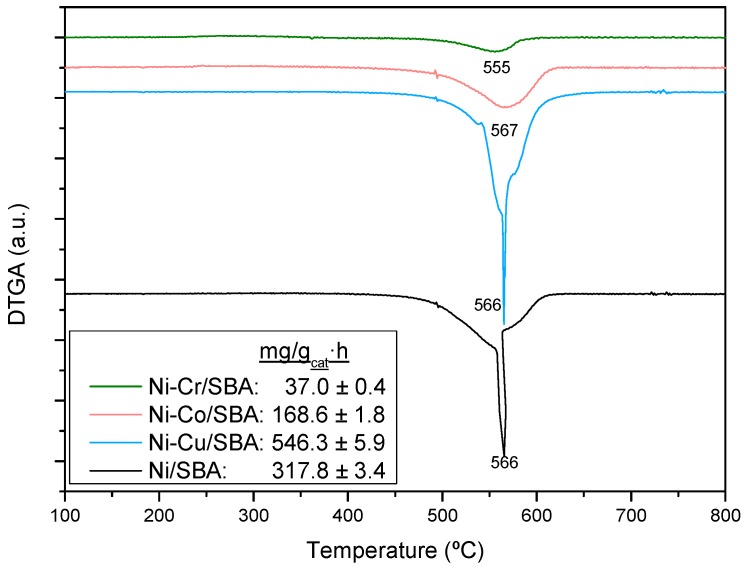
Derivative thermogravimetric analyses (airflow) of the Ni-M/SBA-15 catalysts used in the reforming of model bio-oil aqueous fraction.

The derivative thermograms of the used catalysts are displayed in Figure 9, together with the coke formation rate measured by TGA. Similarly to the steam reforming of the model compounds, the coking rate varies in the order: Ni-Cu/SBA-15 > Ni/SBA-15 > Ni-Co/SBA-15 > Ni-Cr/SBA-15. It must be highlighted the low carbon deposition on the Ni-Cr/SBA-15 catalyst, achieving a reduction of around eight 8 times compared to bare Ni/SBA-15 material. As expected, the carbon deposition is higher than that measured in the model compounds’ steam reforming, due to the low water content in the bio-oil aqueous fraction, which implies unfavoured gasification of the C* intermediate species. On the other hand, the DTG profiles in Figure 8 show that the oxidation of coke takes place between 450 and 625 °C, indicating that carbon fibres were formed over the catalysts. Except for the Ni-Cr/SBA-15 sample, T_max_ are above 565 °C, indicative of high condensation degree of the carbon deposits, which have been reported as the cause of Ni blocking [63]. Formation of highly condensed coke would be favoured by low S/C ratio, as a consequence lower carbon gasification rate at the expense of further dissociation of hydrocarbon intermediate species into carbon atoms. As a result, deactivation could be observed in the Ni-Cu/SBA-15 and Ni/SBA-15 samples within 5 h TOS. Catalytic activity and stability are significantly affected by the Ni-Cu composition. Under dry methane reforming conditions at 600 °C, Song et al. [38] reported that Ni-Cu/Mg(Al)O catalysts with Cu/Ni ratios between 0.25 and 0.5 showed high activity and stability due to the formation of a Ni-Cu alloy, whereas those samples with lower and higher Cu/Ni ratios suffered from rapid deactivation because the nature of coke deposition varies significantly with Ni-Cu composition. In our case, Ni-Cu/SBA-15 (Cu/Ni = 0.25) catalyst used in the steam reforming of bio-oil model compounds may be far from the Cu/Ni ratio necessary to reduce coke deposition over Ni/SBA-15. In the case of the Ni-Co/SBA-15 catalyst, the fact that Co is also active in reforming reactions implies the higher active surface needed to be blocked before noticing deactivation. In addition, although the type of coke has a role in deactivation, high carbon deposition can lead to loss of the support porosity, blocking of pores and loss of the active surface inside the support pores.

The beneficial effect in coke resistance by Cr addition to Ni/SBA-15 catalyst can be ascribed to the small Ni crystallites, since the smaller the crystals, the more the initiation of carbon nucleation is impeded [67]. In addition, Cr addition to Ni catalysts below certain loadings has also been suggested to rearrange the Ni crystal plane and alter Ni electronic properties in such a way that the encapsulation of nickel by inactive carbon filaments is reduced [45].

## 3. Experimental

### 3.1. Catalyst Synthesis and Characterization

A series of catalysts were prepared to study the effect of the addition of Cu, Co and Cr to Ni/SBA-15 catalyst (Ni: 15wt %). The synthesis of SBA-15 was carried out by the hydrothermal method as described by Zhao et al. [68] with air calcination at 550 °C in static conditions for 5 h at a heating rate of 1.8 °C/min carried out to eliminate the template agent. Catalysts are denoted as Ni-M/SBA-15, where M are Cu, Co or Cr. The loading of the second metal in modified catalysts is 4 wt %. Both Ni and the second metal were incorporated by incipient wetness co-impregnation of the corresponding nitrates precursors on SBA-15 support, with subsequent calcination at 600 °C for 5 h with a heating rate of 1.8 °C/min. Calcination temperature was selected after TG analyses of the impregnated materials, in order to guarantee the decomposition of the corresponding precursors during the calcination step.

Catalysts were characterized by inductively coupled plasma atomic emission spectroscopy (ICP-AES), X-ray powder diffraction (XRD), hydrogen temperature programmed reduction (TPR) and nitrogen physisorption analysis (N_2_-BET).

ICP-AES technique was used to determine the metal content in the catalysts. Analyses of the samples, previously dissolved by acidic digestion with H_2_SO_4_ and HF, were performed on a Varian VISTA-PRO AX CCD simultaneous ICP-AES apparatus.

XRD was used to determine the supports porous mesostructure and catalysts’ crystalline phases (according to the Joint Committee on Powder Diffraction Standards—JCPDS index), as well as the mean metallic crystallite diameters calculated by Scherrer equation. Data were acquired on a Philips X’Pert PRO diffractometer, using Cu Kα radiation, a 2 θ increment step of 0.020° and a collection time of 2 s. 

TPR was used to analyze the reducibility of the samples and obtain information about metal-support interaction. Measurements of the samples were performed on a Micromeritics AUTOCHEM 2910, placing the catalyst in a fixed-bed quartz tube under 10% H_2_ in argon flow (35 mL/min) with a heating rate of 5 °C/min from 25 to 900 °C. Effluent gas is forced to flow through a cold trap to remove water produced before reaching the thermal conductivity detector (TCD). Samples were previously degasified under dry argon flow (35 mL/min) at 110 °C for 30 min with a heating rate of 15 °C/ min. 

Textural properties were measured by N_2_ adsorption/desorption analyses at 77 K. using a Micromeritics TRISTAR 3000 sorptometer. Prior to the nitrogen-adsorption, samples were outgassed under vacuum at 200 °C for 4 h. Surface areas were calculated according to BET method. 

### 3.2. Catalytic Tests

**Figure 10:**
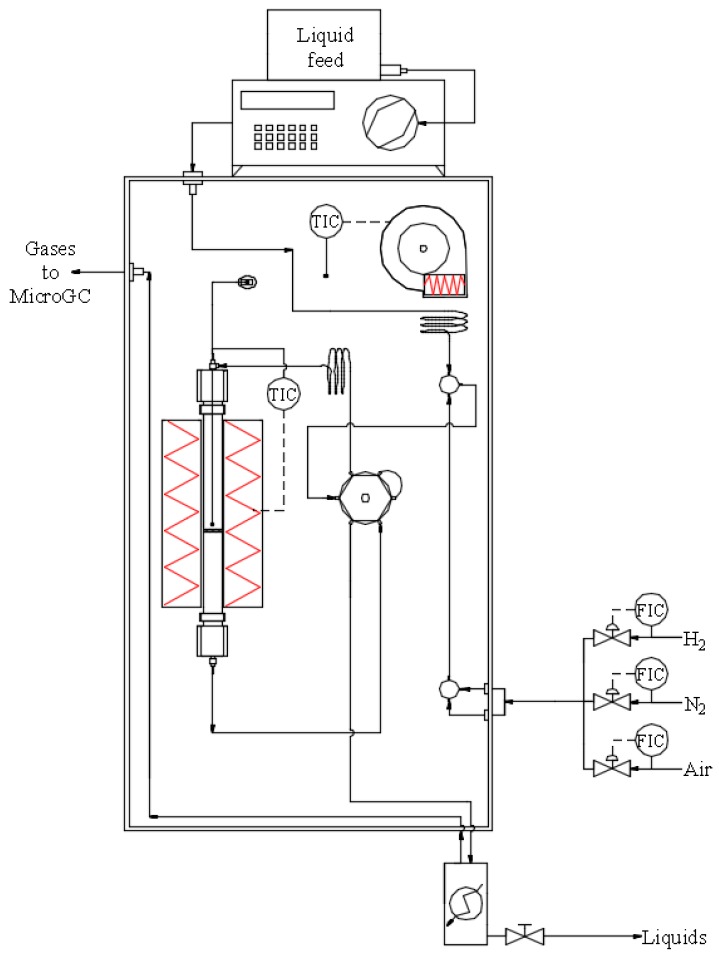
Schematic diagram of the catalytic testing setup [49].

The experimental installation where steam reforming tests were accomplished was a MICROACTIVITY-PRO unit (PID Eng&Tech. S.L.), which consists of a fixed-bed tubular reactor in stainless steel 316 (i.d. = 9.2 mm, L = 300 mm) located inside an electric oven of low thermal inertia, where temperature inside the catalytic bed is measured by means of a thermocouple. All the components are placed inside a hot box in stainless steel 316 with a convector of hot air at 200 °C to prevent the condensation of volatile products in the pipes and to preheat the reactants efficiently. The liquid reaction mixture is fed by means of a GILSON 307 piston pump (0.01–5 mL/min, 400 bar) and passing through an evaporator before mixing with the preheated carrier gas (N_2_) and entering the reactor. At the reactor outlet, there is a thermoelectric unit to condense and separate condensable vapors. A schematic diagram of this setup is shown in Figure 10.

The catalyst (300 mg, undiluted with inert material) was placed inside the reactor. Before testing, catalyst was in situ reduced under flowing pure hydrogen (30 mL/min) at 700 °C for 6.5 h with a heating rate of 2 °C/min. After the catalyst activation, the reaction temperature was fixed for each experiment at 600 °C and catalytic testing was carried out isothermally at atmospheric pressure under nitrogen-diluted conditions. A liquid reaction mixture of the corresponding model compound(s) (acetic acid, hydroxyacetone, furfural and/or phenol) and water was introduced at a flow rate of 0.075 mL/min, vaporized at 200 °C and further eluted by N_2_ (60 mL/min). These conditions were determined in previous experiments to ensure the absence of internal and external diffusion limitations.

In the case of the reforming of acetic acid (AA) and hydroxyacetone (HA), a water/compound molar ratio = 8 was used. In the case of the reforming of phenol (Ph) and furfural (Fur), since their solubility in water is limited (82–83 g/ at 20 °C), a water/compound molar ratio = 66 was used in order to guarantee homogeneous mixture. In addition, the steam reforming of a simulated bio-oil aqueous fraction was performed. The composition of the mixture was based on the aqueous fraction obtained in the catalytic fast-pyrolysis of partially de-ashed wheat straw using H-ZSM-5 [16], where bio-oil phases separation occurs without any water addition. In that investigation, carboxylic acids, ketones-ethers, furans and oxygenated aromatics were identified as the major compounds groups in aqueous fractions, accounting for the 85 wt % of the organic components, and the water content was 41 wt % (aqueous fraction composition not published; the composition was provided by the authors of the work [16] in the context of the RESTOENE 2 project). The most representative compound of each of the above-mentioned groups were selected. Thus, the mixture composition was 31 wt % acetic acid, 18 wt % hydroxyacetone, 6 wt % furfural and 4 wt % phenol in water.

The composition of the output gas stream was determined online in an Agilent 490 Micro-GC equipped with a Pora Plot U column (10 m), a Molecular Sieve 5A column (20 m) and thermal conductivity Detectors (TCD). Condensable vapors were trapped in the condenser at 4 °C and analyzed in the chromatograph Varian CP-3900 equipped with a CP-WAX 52 CB (30 m × 0.25 mm, DF = 0.25) column and flame ionization detector (FID). 

Reactants conversion and products selectivity were calculated as follows:

(22) $$X\left(\%\right)=\frac{F_{reactant,in}-F_{reactant,out}}{F_{reactant,\;in}}\cdot 100$$

(23) $$S_{i}\left(mol\%\right)=\frac{F_{i,out}}{\sum F_{i,out}}\cdot 100$$

where *F_reactant,in_* and *F_reactant,out_* are the molar flow rates of the corresponding reactant (acetic acid, hydroxyacetone, furfural or phenol) at the inlet and outlet of the reactor, respectively. *F_i,out_* is the molar flow rate of the product *i* species (hydrogen, carbon dioxide, carbon monoxide and methane) at the outlet of the reactor. The products outlet stream was analyzed several times along the reaction time (time-on-stream = 5 h). In all experiments, the composition remained constant and thus the given reaction parameters are the averaged values.

Carbon deposited during reaction was evaluated through characterization of the used catalyst by thermogravimetric analyses (TGA). Measurements were performed in air flow on a TA instruments SDT 2960 thermobalance, with a heating rate of 5 °C/min up to 800 °C. The value of coke deposition is given as:

(24) $$C_{dep}\left(wt\%\right)=\frac{m_{coke}}{m_{used\;catalyst}}\cdot 100$$

### 3.3. Thermodynamic Calculations

The thermodynamic equilibrium of a closed system with specific temperature and pressure is reached when the total Gibbs energy reaches a minimum. Thereby, the equilibrium composition of the outlet stream from the steam reforming of the model compounds (experimental feed compositions) was predicted at atmospheric pressure and different temperatures (300–1000 °C) using the minimization of the Gibbs free energy method (Gibbs reactor) in Aspen HYSYS v10 simulation software. This software includes chemical component libraries as well as thermodynamic property prediction methods. Adding the compounds expected at the outlet gas stream is already enough to determine the equilibrium composition without the necessity of indicating a reaction set. Using the directions given by the software and according to other authors [17,69,70], Peng–Robinson–Stryjek–Vera (PRSV) thermodynamic method was selected. 

## 4. Conclusions

The Ni-M/SBA-15 catalysts (M: Cu, Co, Cr) prepared for this work are active in the steam reforming of the compounds typically contained in the bio-oil aqueous fractions, reaching high hydrogen production. However, while Ni-Co/SBA-15 and Ni-Cr/SBA-15 achieved conversions above 96% in all cases, Ni/SBA-15 and Ni-Cu/SBA-15 samples suffered from deactivation. A relationship between loss of activity and coke formation rate could be established, although the nature of coke deposits also has an important role. The higher activity of Ni-Co/SBA-15 and Ni-Cr/SBA-15 has been ascribed to the presence of Co as an additional active metal and to the small Ni crystallites and strong interaction with the support in the Ni-Cr/SBA-15 sample.

When these catalysts were tested in the steam reforming of single model compounds (acetic acid, hydroxyacetone, furfural or phenol), a decrease in reactivity with increasing carbon number could be inferred, although higher S/C ratio promoted higher conversion in the steam reforming of furfural and phenol. The main products’ (H_2_, CO_2_, CO and CH_4_) distribution, which slightly differs from the thermodynamic prediction by Gibbs energy minimization, could be explained by the complex reaction network implied in the process. A competition between hydrogenation and oxidation of carbon intermediate species will determine the product distribution. Catalysts with high capacity for H_2_O activation during the oxidation of these carbon intermediates will lead to higher H_2_ production by steam reforming, reducing CH_4_ formation.

The coking rate achieved in the steam reforming of model compounds varied in the order hydroxyacetone > acetic acid > furfural > phenol, as a consequence of the nature of the reactant (carbon number and aromaticity) and the S/C ratio, but deactivation also depends on the nature of coke. In this sense, a more graphitic coke was formed in the phenol steam reforming, while a poorly evolved coke was predominant with furfural.

In the steam reforming of a surrogate of the aqueous fraction obtained in the catalytic fast-pyrolysis of de-ashed wheat straw, with S/C lower than stoichiometric, the superior catalytic behavior of Ni-Co/SBA-15 and Ni-Cr/SBA-15 was highlighted. However, Ni/SBA-15 and Ni-Cu/SBA-15 had worse behavior because both deactivated. In addition, the coking rate varied in the order: Ni-Cu/SBA-15 > Ni/SBA-15 > Ni-Co/SBA-15 > Ni-Cr/SBA-15. The low coke deposition on the Ni-Cr/SBA-15 catalyst (~8 times lower than Ni/SBA-15) is attributed to small Ni crystallites avoiding the formation of the intermediary surface species of coke deposition, which also led to poorly condensed coke. On the contrary, the predominance of aged coke with high graphitization degree has been identified as the cause for the loss of conversion of Ni-Cu/SBA-15 sample.

## Figures and Tables

**Table 1:** Physicochemical properties of the fresh catalysts.

Sample	Ni^a^ (wt %)	M^a,b^ (wt %)	S_BET_ (m^2^g^−1^)	V_pore_^c^ (cm^3^g^−1^)	D_pore_^d^ (nm)	D_Ni_^e^ (nm)	TPR Profile < 400 °C	TPR Profile > 400 °C
							Area (%)	Area (%)
Ni/SBA-15	13.5 ± 0.1	-	521 ± 4	0.77 ± 0.02	8.3 ± 0.1	10.6 ± 0.5	69.8	30.2
Ni-Cu/SBA-15	15.0 ± 0.1	4 ± 0.1	485 ± 1	0.71 ± 0.01	8.1 ± 0.1	9.9 ± 0.2	99.0	1.0
Ni-Co/SBA-15	14.5 ± 0.1	4 ± 0.1	486 ± 1	0.72 ± 0.02	8.0 ± 0.1	9.0 ± 0.3	67.7	32.3
Ni-Cr/SBA-15	14.3 ± 0.1	3.6 ± 0.1	482 ± 3	0.68 ± 0.03	8.3 ± 0.1	5.8 ± 0.1	16.5	83.5

^a^ Measured by Inductively Coupled Plasma Atomic Emission Spectroscopy (ICP-AES); ^b^ M: Second metal (Cu, Co or Cr); ^c^ Determined at P/P_0_ = 0.97; ^d^ Maximum of the BJH (Barrett, Joyner and Halenda) pore size distribution; ^e^ Calculated from the (1 1 1) reflection of Ni in XRD of reduced catalysts.

**Table 2:** Coke formation rate (mg/g_cat_·h) in the steam reforming of hydroxyacetone (HA), acetic acid (AA), phenol (Ph) and furfural (Fur) using the Ni-M/SBA-15 catalysts.

Catalyst	AA	HA	Fur	Ph
Ni/SBA-15	44.8 ± 0.5	96.3 ± 1.0	21.2 ± 0.2	14.1 ± 0.2
Ni-Cu/SBA-15	154.0 ± 1.6	165.0 ± 1.8	96.7 ± 1.1	19.8 ± 0.2
Ni-Co/SBA-15	35.8 ± 0.4	77.0 ± 0.8	9.2 ± 0.1	9.0 ± 0.2
Ni-Cr/SBA-15	3.5 ± 0.1	18.3 ± 0.2	2.6 ± 0.1	7.0 ± 0.2

**Table 3:** Conversion (mol %) of model compounds in the reforming of bio-oil aqueous fraction surrogate using the Ni-M/SBA-15 catalysts at time-on-stream (TOS) = 5 h.

Catalyst	AA	HA	Fur	Ph
Ni/SBA-15	87.6 ± 1.4	99.5 ± 0.0	77.4 ± 3.0	69.9 ± 2.6
Ni-Cu/SBA-15	80.4 ± 2.2	96.5 ± 0.4	84.7 ± 2.1	74.0 ± 2.3
Ni-Co/SBA-15	99.5 ± 0.1	100 ± 0.0	97.9 ± 0.3	89.5 ± 0.9
Ni-Cr/SBA-15	98.4 ± 0.2	99.9 ± 0.0	95.5 ± 0.6	91.5 ± 0.7
